# 

**Published:** 1916-03-18

**Authors:** 


### CONTENTS.

| | PAGE |
|---|---|
| **Secret Terms in Physicians' Prescriptions** .. | 535 |
| **The Meaning of "Infectious"** ... ... ... | 536 |
| **Hospital and Institutional News** ... ... ... | 537 |
| The Queen Visits the Florence Nightingale Hospital—Lord Knutsford—A Seasonable Birthday Gift—Another Brownlow Hill Infirmary Scandal—A Remarkable Episode in Hospital Finance—The Hospital Sculptor—A Batch of War Magazines—A Doctor's Conscientious Objection—The "Fiction" of Puerperal Fever —The Importance of Trivial Illness—The Hope of the Cripple—The Discipline of Patients in Military Hospitals—A Historical Pamphlet—An Isolation Hospital Without a Water Supply—Hospital for Limbless Warriors, Glasgow—Sir Alfred Jones Memorial Hospital, Garston—A Hospital Secretary as Major—A Zeppelin Ward—The West Ham and Eastern General Hospital—Sir F. Milner and the Perth Hospitals—The East Sussex Tuberculosis Scheme: Successful Opposition—Aberdeen Open-Air Children's Ward—Tuberculosis Patients—Sanatorium Benefit in London—Economical School Inspection in Cornwall—Increased Cost of Maintenance—Deaths by Starvation or Privation—The Luxury of Patent Medicines—This Week's Drug Market. | |
| **Is Goitre an Infective Disease?** ... ... ... | 543 |
| A Revolutionary Hypothesis and its Consequences. | |
| **Hospitals and the Spirit Tax** ... ... ... | 544 |
| **A Famous Foundling Hospital** ... ... ... | 545 |
| Vospitatel'nie Dom, Moscow. | |
| **The "Dreadnought" Hospital. A Successful and Busy Year** ... ... ... ... ... | 546 |
| **Hospitals and the Poets** ... ... ... ... | 547 |
| VI.—Walt Whitman as a Hospital Orderly. | |
| **The Matrons' and Sisters' Department** ... | 549 |
| Round the Hospitals. | |
| Sisters and Ward Economy—Hospital Manners —Thrift Bonuses—A Matron and her Laundry —Whist Drives for the Wounded—Mortuary Equipment. | |
| **Textile Supplies for Hospitals** ... ... ... | 551 |
| VII.—Wool Clothing. | |
| **Macmillan and Co. (Limited) and Another v. The Nursing Press (Limited) and Others** | 553 |
| (Before Mr. Justice Ridley and a Special Jury.) The Judge's Summing-up. | |
| **Passing Events and Topics** ... ... ... ... | 556 |
| **The Editor's Letter-Box** ... ... ... ... | 557 |
| Cancer and the Scientific Spirit. | |
| **Medical Appointments** ... ... ... ... | 557 |
| **Parliament and the Profession** ... ... ... | 558 |
| **Gifts and Bequests** ... ... ... ... ... ... | iv |
| **Matrons' and Sisters' Appointments** ... ... | iv |
| **Personalia**... ... ... ... ... ... ... ... | iv |
| **Annual Meetings** ... ... ... ... ... | iv |
| **The Book Market** ... ... ... ... ... | iv |
| **The Institutional Worker** .. .. (Suppl.) | 1 |
| Economies in the Matron's Department: Answers to February Question (No. 2). | |

